# Electrocardiographic Ventricular Repolarisation Parameters in Morbidly Obese Individuals

**DOI:** 10.3390/medicina62101925

**Published:** 2026-10-06

**Authors:** İlker Kızmaz, Erdinç Yaman, Gizem Nur Sözen, Betül Danapınar, Mehmet Saydam, Süleyman Baş, Kadem Arslan

**Affiliations:** 1 Department of Internal Medicine, Mersin Mut State Hospital, 33600 Mersin, Turkey; 2 Department of Internal Medicine, Tekirdağ Kapaklı State Hospital, 59510 Tekirdağ, Turkey; 3 Department of Internal Medicine, Hakkari Şemdinli State Hospital, 30800 Hakkari, Turkey; 4 Department of Internal Medicine, Karabük University Education and Research Hospital, 78200 Karabük, Turkey; 5 Department of General Surgery, Lokman Hekim University, Lokman Hekim Istanbul Hospital, 34912 Istanbul, Turkey; 6 Department of Internal Medicine, University of Health Sciences, Sancaktepe Sehit Prof. Dr. Ilhan Varank Training and Research Hospital, 34785 Istanbul, Turkey

**Keywords:** morbid obesity, electrocardiography, ventricular repolarisation, QT interval, Tp-e interval, iron metabolism

## Abstract

*Background and Objectives*: Obesity may adversely affect cardiovascular function and cardiac electrical activity. This study aimed to evaluate electrocardiographic indices of ventricular repolarisation in morbidly obese individuals compared with non-obese controls. *Materials and Methods*: This retrospective, single-centre study included 112 morbidly obese individuals with a body mass index >40 kg/m^2^ and 51 non-obese controls. QT and Tp-e intervals and Tp-e/QT ratios were measured from resting ECG recordings. Heart-rate-corrected QT intervals were calculated using the Bazett and Fridericia formulae. Between-group comparisons, multivariable regression analyses, sensitivity analyses, and exploratory analyses of iron metabolism parameters were performed. Multiple comparisons were adjusted using the Benjamini–Hochberg false discovery rate (FDR) procedure. *Results*: Morbidly obese individuals had a shorter RR interval (*p* = 0.020) and higher QTc-Bazett (*p* = 0.016) and Tp-e/QT (*p* = 0.030) than controls; however, these differences did not remain significant after FDR correction (all adjusted *p* = 0.080). QTc-Fridericia did not differ between groups (*p* = 0.240). After multivariable adjustment, morbid obesity was not significantly associated with Tp-e/QT (*p* = 0.338) or QTc-Fridericia (*p* = 0.177). A sensitivity analysis restricted to morbidly obese individuals without recorded comorbidities similarly showed no significant differences in ventricular repolarisation indices after FDR correction. Within the morbidly obese group, men had higher Tp-e-based indices than women after FDR correction. Exploratory analyses showed associations between iron metabolism parameters and Tp-e-based indices, with ln-ferritin remaining positively associated with Tp-e/QT after adjustment (*p* = 0.012). *Conclusions:* The present findings did not demonstrate a consistent association between morbid obesity alone and altered ventricular repolarisation, as the observed between-group differences were not robust across multiple-comparison correction, adjusted analyses, and sensitivity analyses. Sex-related differences and exploratory associations with iron metabolism suggest a multifactorial influence on ventricular repolarisation in morbid obesity. These findings warrant prospective validation using direct arrhythmic outcomes.

## 1. Introductıon

Obesity is a chronic, complex, and multifactorial condition that adversely affects health and is associated with numerous comorbidities, including type 2 diabetes, hypertension, dyslipidaemia, cardiovascular and cerebrovascular diseases, obstructive sleep apnoea syndrome, non-alcoholic fatty liver disease, osteoarthritis, polycystic ovary syndrome, depression, and several malignancies. In addition to its substantial impact on individual health, obesity imposes a considerable burden on healthcare systems [1].

The cardiovascular effects of obesity involve multiple haemodynamic, metabolic, and neurohumoral mechanisms. Adipose tissue-derived mediators, including leptin, have been associated with cardiovascular alterations [2]. Obesity is also accompanied by increases in total blood volume and stroke volume and may be associated with increased sympathetic activity, heart rate, and arterial blood pressure [2]. Chronic haemodynamic loading can contribute to left ventricular remodelling and hypertrophy, while increased filling pressures and left atrial enlargement may promote atrial structural and electrical abnormalities [2,3]. Beyond these structural changes, obesity has been associated with alterations in cardiac electrophysiology and an increased risk of sudden cardiac death [4]. Ventricular repolarisation abnormalities have therefore been proposed as one of several potential mechanisms linking obesity with adverse cardiac outcomes, and alterations in electrocardiographic ventricular repolarisation indices have been reported in obese populations [5].

Surface electrocardiography provides several non-invasive indices for the assessment of ventricular repolarisation. In addition to the QT and heart-rate-corrected QT (QTc) intervals, the T-wave peak-to-end (Tp-e) interval and the Tp-e/QT and Tp-e/QTc ratios have been used as indices of ventricular repolarisation and repolarisation heterogeneity [6,7,8]. However, these ECG-derived parameters are surrogate electrophysiological indices and should not be considered direct measures of clinical arrhythmic risk. Moreover, interpretation of QTc may be influenced by the method used for heart-rate correction, particularly when heart rates differ between populations [9].

Against this background, the primary aim of this study was to compare electrocardiographic ventricular repolarisation indices, including QT, QTc, Tp-e, Tp-e/QT, and Tp-e/QTc, between morbidly obese individuals and non-obese controls. In addition, we explored sex-related differences and the associations between iron metabolism parameters and ventricular repolarisation indices within the morbidly obese group.

## 2. Materials and Methods

### 2.1. Study Group

This retrospective study included patients who had presented to the Internal Medicine or General Surgery outpatient clinics at SBÜ Sancaktepe Şehit Prof. Dr. İlhan Varank Training and Research Hospital prior to bariatric surgery between 1 March 2023 and 1 March 2024. The study included 112 morbidly obese individuals aged between 18 and 65 years, with a body mass index (BMI) > 40 kg/m^2^ and a pre-operative resting electrocardiogram (ECG) recording.

Individuals under 18 or over 65 years of age, pregnant women, patients with a BMI < 40 kg/m^2^, and those without a pre-operative resting ECG recording were excluded from the study. Patients with a history of hyperthyroidism, valvular heart disease, heart failure, coronary artery disease, anaemia, acute infection or active inflammatory disease, as well as those receiving antiarrhythmic medication, were also excluded because these conditions or treatments could potentially affect ventricular repolarisation.

The control group comprised 51 individuals who attended outpatient clinics at the same centre and during the same time period, who did not have a diagnosis of obesity, who had no known cardiovascular disease or systemic disease that could cause arrhythmogenic effects, who were not on regular medication, and who had a resting ECG recording available. The control group was selected to have similar characteristics to the morbidly obese group in terms of age and sex.

### 2.2. Data Collection

Demographic characteristics, anthropometric measurements, comorbidities, medication use, and available biochemical and haematological parameters were retrospectively obtained from the hospital information system (HIS). Body mass index was calculated as body weight divided by height squared (kg/m^2^). Resting ECG recordings were retrieved for electrocardiographic analysis.

### 2.3. Electrocardiographic Measurements

Standard 12-lead ECGs were obtained using a Schiller Cardiovit AT-102 G2 electrocardiograph (Schiller AG, Switzerland) at a paper speed of 25 mm/s and an amplitude of 10 mm/mV. ECG recordings not available in digital format were digitised using a high-resolution scanner, and interval measurements were performed on the digital ECG images using AutoCAD 2021 (Autodesk, Inc., San Francisco, CA, USA) software. Electrode placement was performed by trained personnel according to standard anatomical landmarks.

Electrocardiographic measurements were performed using lead V6. For each participant, three consecutive cardiac cycles with a stable baseline were evaluated, and the mean of the three measurements was used for analysis. The QT interval was measured from the onset of the QRS complex to the end of the T wave, the Tp-e interval from the peak to the end of the T wave, and the RR interval between two consecutive R waves.

The QT interval was corrected for heart rate using both Bazett’s formula (QTc-Bazett = QT/√RR) and Fridericia’s formula (QTc-Fridericia = QT/RR^(1/3)), with RR expressed in seconds for QT correction. The Tp-e/QT ratio was calculated, and Tp-e/QTc ratios were calculated separately using Bazett- and Fridericia-corrected QT intervals (Tp-e/QTc-Bazett and Tp-e/QTc-Fridericia, respectively). All electrocardiographic measurements were performed by the same investigator independently of the participants’ clinical data.

To assess intraobserver reproducibility, QT and Tp-e measurements were repeated by the same investigator in a randomly selected subset of 24 ECG recordings, comprising 12 morbidly obese individuals and 12 controls, using the same measurement protocol as in the original assessment. For the repeat assessment, the ECG recordings were provided without participant names or study identification codes, and the investigator had no access to the original QT and Tp-e measurement values. Intraobserver reliability was evaluated using a two-way mixed-effects, absolute-agreement, single-measure intraclass correlation coefficient (ICC) with 95% confidence intervals. Agreement between the original and repeat measurements was additionally assessed using Bland–Altman analysis.

### 2.4. Ethics Committee Approval

This study was approved by the Clinical Research Ethics Committee of SBÜ Sancaktepe Şehit Prof. Dr. İlhan Varank Training and Research Hospital (Decision No: 2024/83, Date: 13 March 2024). The study employed a retrospective design and was conducted in accordance with the principles of the Declaration of Helsinki.

### 2.5. Statistical Analysis

Statistical analyses were performed using IBM SPSS Statistics for Windows, Version 25.0 (IBM Corp., Armonk, NY, USA). The distribution of continuous variables was assessed using the Kolmogorov–Smirnov and Shapiro–Wilk tests. Normally distributed continuous variables were presented as mean ± standard deviation, whereas non-normally distributed continuous variables were presented as median [interquartile range]. Categorical variables were expressed as number and percentage [n (%)].

For comparisons between two independent groups, the independent-samples *t* test was used for normally distributed continuous variables and the Mann–Whitney U test for non-normally distributed continuous variables. Categorical variables were compared using the chi-square test. Correlations between continuous variables were assessed using Pearson’s correlation coefficient when parametric assumptions were met and Spearman’s rank correlation coefficient otherwise.

Both Bazett- and Fridericia-corrected QT intervals were included in the analyses to evaluate the robustness of the findings to the method of heart-rate correction. Effect sizes were reported as Hedges’ g for comparisons performed using the independent-samples *t* test and as rank-biserial correlations (r_rb) for comparisons performed using the Mann–Whitney U test. Percentile bootstrap 95% confidence intervals for effect sizes were estimated using 10,000 resamples performed independently within groups with replacement.

Intraobserver reproducibility of QT and Tp-e measurements was assessed using a two-way mixed-effects, absolute-agreement, single-measure intraclass correlation coefficient (ICC) with 95% confidence intervals. Agreement between the original and repeat measurements was additionally evaluated using Bland–Altman analysis, with mean differences and 95% limits of agreement calculated for each parameter.

To assess the potential influence of differences in comorbidity burden between the groups, a sensitivity analysis was performed by restricting the morbidly obese group to individuals without recorded comorbidities and comparing them with the healthy control group using the same statistical approach as in the primary between-group analysis.

Multivariable linear regression analyses were performed to evaluate the associations of morbid obesity with Tp-e/QT and QTc-Fridericia after adjustment for age, sex, magnesium, potassium, creatinine, and calcium. These covariates were selected during the analytical process based on clinical and electrophysiological considerations, including the recognised influence of age and sex on ventricular repolarisation, the direct electrophysiological relevance of potassium, magnesium, and calcium, and the potential influence of renal function, represented by creatinine, on the metabolic and electrolyte milieu. An obesity-by-sex interaction term was additionally examined to assess whether the association between obesity and Tp-e/QT differed according to sex. A sensitivity analysis was also performed by including the RR interval in the Tp-e/QT multivariable model to assess the potential influence of heart-rate-related differences. Regression assumptions were assessed using residual distributions, normal probability plots, residual-versus-predicted plots, collinearity diagnostics, and Cook’s distance.

Associations between iron metabolism parameters and ECG indices were evaluated as exploratory analyses. Spearman’s correlation analysis was used for these associations, except for the relationship between TIBC and RR interval, for which Pearson’s correlation analysis was applied based on the distributional characteristics of the variables. The Benjamini–Hochberg FDR procedure was applied across the 32 iron–ECG correlation tests. Following these exploratory analyses, the ferritin–Tp-e/QT association was selected post hoc for adjusted modelling because it had the largest correlation coefficient among the FDR-significant ferritin-related associations. Because ferritin showed a right-skewed distribution, ferritin values were natural-log transformed before regression analysis. The association between ln-ferritin and Tp-e/QT was subsequently evaluated using multivariable linear regression adjusted for age, sex, NLR, and glucose. This analysis was considered exploratory and hypothesis-generating rather than confirmatory.

To account for multiple comparisons, the Benjamini–Hochberg false discovery rate (FDR) procedure was applied separately to the primary between-group ECG comparisons, the comorbidity-restricted sensitivity analysis, exploratory sex-based ECG comparisons, and exploratory iron–ECG correlation analyses. All tests were two-sided. Statistical significance was defined as *p* < 0.05; for analyses subjected to multiplicity correction, statistical significance was based on an FDR-adjusted *p* < 0.05.

## 3. Results

The demographic and anthropometric characteristics of the morbidly obese group and the control group are shown in Table 1. No statistically significant difference was observed between the two groups in terms of age and sex distribution (*p* > 0.05). Similarly, no significant difference was observed between the groups in terms of height (*p* > 0.05). The body weight and body mass index (BMI) values of the morbidly obese group were statistically significantly higher than those of the control group (***p* < 0.001, *p* < 0.001**) (Table 1).

It was found that 59.8% of morbidly obese patients (n = 67) had at least one comorbidity. The most common comorbidity was hypertension (31.2%), followed by diabetes mellitus (17%) and dyslipidaemia (11.6%). In addition, hypothyroidism and asthma/COPD/obstructive sleep apnoea syndrome (OSAS) were each present in 8.9% of patients (Table 2).

A comparison of the biochemical parameters between the morbidly obese group and the control group is shown in Table 3. Glucose and magnesium levels in the morbidly obese group were found to be statistically significantly higher than those in the control group (*p* < 0.001, *p* = 0.023). Furthermore, white blood cell and neutrophil counts in the morbidly obese group were significantly higher than those in the control group (*p* = 0.001, *p* < 0.001). However, no statistically significant differences were observed between the groups in terms of lymphocyte and haemoglobin levels (*p* > 0.05). The neutrophil-to-lymphocyte ratio (NLR) in the morbidly obese group was found to be statistically significantly higher than that in the control group (*p* < 0.001) (Table 3).

A comparison of electrocardiographic ventricular repolarisation parameters between the morbidly obese and control groups is presented in Table 4. In the nominal comparisons, the morbidly obese group had a shorter RR interval (*p* = 0.020), higher QTc-Bazett (*p* = 0.016), and higher Tp-e/QT ratio (*p* = 0.030) than the control group. However, none of these differences remained statistically significant after Benjamini–Hochberg false discovery rate correction (all FDR-adjusted *p* = 0.080). The corresponding effect sizes were modest (Hedges’ g = −0.397 for RR, r_rb = 0.236 for QTc-Bazett, and r_rb = 0.212 for Tp-e/QT). No between-group differences were observed for QT, QTc-Fridericia, Tp-e, Tp-e/QTc-Bazett, or Tp-e/QTc-Fridericia (all FDR-adjusted *p* > 0.05) (Table 4). Furthermore, in a multivariable linear regression analysis adjusted for age, sex, magnesium, potassium, creatinine, and calcium, morbid obesity was not significantly associated with QTc-Fridericia (B = 7.246 ms, 95% CI −3.307 to 17.799; *p* = 0.177).

Intraobserver reproducibility was assessed for both QT and Tp-e measurements. The single-measure ICC was 0.997 for QT (95% CI 0.992–0.999) and 0.997 for Tp-e (95% CI 0.992–0.999), indicating a high level of intraobserver agreement. Bland–Altman analysis showed mean differences of −1.10 ms for QT (95% limits of agreement, −5.07 to 2.87 ms) and −0.22 ms for Tp-e (95% limits of agreement, −2.05 to 1.62 ms).

To further assess the potential influence of differences in comorbidity burden between the study groups, a sensitivity analysis was performed by restricting the morbidly obese group to individuals without recorded comorbidities (n = 45) and comparing them with healthy controls (n = 51). In this restricted analysis, the morbidly obese subgroup had a shorter RR interval than the control group (721.50 ± 138.53 vs. 781.15 ± 109.93 ms; raw *p* = 0.021; Hedges’ g = −0.477, 95% CI −0.939 to −0.072); however, this difference did not remain statistically significant after FDR correction (FDR-adjusted *p* = 0.168). No statistically significant differences were observed for QT, QTc-Bazett, QTc-Fridericia, Tp-e, Tp-e/QTc-Bazett, Tp-e/QTc-Fridericia, or Tp-e/QT (all FDR-adjusted *p* > 0.05) (Table 5). The differences in QTc-Bazett and Tp-e/QT observed in the primary between-group comparison were not statistically significant in this restricted analysis.

In the exploratory sex-based analysis within the morbidly obese group, Tp-e, Tp-e/QTc-Bazett, Tp-e/QTc-Fridericia, and Tp-e/QT were higher in males than in females. These differences remained statistically significant after Benjamini–Hochberg false discovery rate correction (FDR-adjusted *p* = 0.031, 0.031, 0.017, and 0.001, respectively), whereas no significant sex-related differences were observed in RR, QT, QTc-Bazett, or QTc-Fridericia after FDR correction (all FDR-adjusted *p* > 0.05) (Table 6). In the multivariable linear regression analysis adjusted for age, sex, magnesium, potassium, creatinine, and calcium, the overall model was statistically significant (F(7,137) = 3.977, *p* = 0.001), explaining 16.9% of the variance in Tp-e/QT (R^2^ = 0.169; adjusted R^2^ = 0.126). Morbid obesity was not significantly associated with the Tp-e/QT ratio after adjustment (B = 0.006, 95% CI −0.006 to 0.018; *p* = 0.338), whereas female sex was significantly associated with a lower Tp-e/QT ratio (B = −0.024, 95% CI −0.039 to −0.010; *p* = 0.001). Age, magnesium, potassium, creatinine, and calcium were not significantly associated with Tp-e/QT (all *p* > 0.05) (Table 7). Furthermore, the obesity-by-sex interaction was not statistically significant (B = −0.002, 95% CI −0.025 to 0.020; *p* = 0.836), indicating no evidence that the association between obesity and Tp-e/QT differed by sex.

In a sensitivity analysis, RR interval was also included in the Tp-e/QT multivariable model to assess the potential influence of heart-rate-related differences. The association between morbid obesity and Tp-e/QT remained statistically non-significant after additional adjustment for RR interval (B = 0.005, 95% CI −0.007 to 0.018; *p* = 0.400). RR interval was also not significantly associated with Tp-e/QT (*p* = 0.595).

Exploratory analyses demonstrated several correlations between iron metabolism parameters and ventricular repolarisation indices after FDR correction (Table 8). TIBC was inversely correlated with Tp-e and Tp-e-based ratios, whereas ferritin showed positive correlations with several Tp-e-based indices. Among the FDR-significant ferritin-related associations, the largest correlation coefficient was observed between ferritin and Tp-e/QT (Spearman’s r = 0.386, FDR-adjusted *p* = 0.001). Transferrin saturation was weakly correlated with Tp-e/QTc-Bazett (Spearman’s r = 0.244, FDR-adjusted *p* = 0.045), while no correlations involving serum iron remained statistically significant after FDR correction. Given the exploratory nature of these analyses, the ferritin–Tp-e/QT association was selected post hoc for adjusted modelling because it had the largest correlation coefficient among the FDR-significant ferritin-related associations. Because ferritin showed a right-skewed distribution, ferritin values were natural-log transformed for multivariable analysis. In an exploratory multivariable linear regression model, ln-ferritin remained positively associated with Tp-e/QT after adjustment for age, sex, NLR, and glucose (B = 0.010, β = 0.265, 95% CI 0.002–0.018; *p* = 0.012). The overall model was statistically significant (R^2^ = 0.172, adjusted R^2^ = 0.131; F(5,102) = 4.227, *p* = 0.002).

## 4. Discussion

In this study, ventricular repolarisation parameters were comprehensively evaluated in morbidly obese individuals and compared with those of non-obese controls. In the unadjusted comparisons, the morbidly obese group had a shorter RR interval and higher Bazett-corrected QT (QTcB) and Tp-e/QT values. However, these differences did not remain statistically significant after correction for multiple comparisons. Moreover, QT corrected using the Fridericia formula (QTcF) did not differ significantly between the groups, and morbid obesity was not significantly associated with either Tp-e/QT or QTcF after adjustment for relevant covariates. A sensitivity analysis restricted to morbidly obese individuals without recorded comorbidities similarly showed no significant between-group differences in ventricular repolarisation indices after FDR correction. In contrast, sex-related differences in several Tp-e-based indices remained significant after multiple-comparison correction, while exploratory analyses also identified associations between selected iron metabolism parameters and ventricular repolarisation indices. Taken together, these findings indicate that the observed between-group differences were modest and not robust across multiple-comparison correction, adjusted analyses, and sensitivity analyses, while their interpretation may also depend on the method of heart-rate correction and individual clinical characteristics. Importantly, as clinical arrhythmic events were not assessed, these ECG-derived findings should be interpreted as differences in surrogate electrophysiological indices rather than as direct evidence of increased arrhythmic risk.

The difference in comorbidity burden between the groups warrants particular consideration, as the control group was comparatively healthy, whereas comorbidities were common among the morbidly obese participants. In the sensitivity analysis restricted to morbidly obese individuals without recorded comorbidities, the differences in QTcB and Tp-e/QT observed in the primary between-group analysis were no longer statistically significant. Although the smaller sample size of this subgroup may have reduced statistical power, these findings suggest that the between-group differences should not be interpreted as reflecting an effect of morbid obesity independent of comorbidity burden.

Previous studies have reported alterations in ventricular repolarisation indices in individuals with obesity, particularly prolongation of QTc and other indices of delayed ventricular repolarisation [4]. Inanir et al. found significantly higher QTc and Tp-e intervals and higher Tp-e/QT and Tp-e/QTc ratios in extremely obese individuals compared with healthy controls [10]. Similarly, Aydın reported reductions in Tp-e, Tp-e/QT, and Tp-e/QTc following substantial weight loss after laparoscopic sleeve gastrectomy in patients with morbid obesity, whereas QT and QTc did not change significantly [5]. In the present study, the higher QTcB and Tp-e/QT values observed in the primary between-group comparisons were broadly consistent with these previous observations. However, these differences did not remain statistically significant after correction for multiple comparisons, and no significant between-group difference was observed for QTcF. Furthermore, morbid obesity was not significantly associated with Tp-e/QT or QTcF in the multivariable analyses. Given the shorter RR interval observed in the morbidly obese group, the potential influence of heart-rate-related differences on Tp-e/QT was further examined by including RR interval in the multivariable model. This did not materially alter the finding, as the association between morbid obesity and Tp-e/QT remained statistically non-significant. These findings suggest that ventricular repolarisation alterations reported in individuals with obesity may not be uniform across different ECG indices and that their interpretation may depend on heart rate, the method used for QT correction, and other clinical characteristics.

Several mechanisms may contribute to alterations in ventricular repolarisation in obesity, including haemodynamic loading, structural cardiac remodelling, autonomic dysfunction, chronic low-grade inflammation, and metabolic abnormalities [4,11,12]. Adipose tissue is metabolically active and produces several adipokines and pro-inflammatory mediators that may influence cardiovascular structure and function [13]. In addition, obesity has been associated with alterations in autonomic balance, which may affect heart rate and ventricular repolarisation [14]. In the present study, the RR interval was shorter in the morbidly obese group in the primary between-group comparison, corresponding to a higher resting heart rate; however, this difference did not remain statistically significant after correction for multiple comparisons. Although autonomic dysfunction represents a plausible mechanism linking obesity with changes in cardiac electrophysiology, sympathetic activity was not directly assessed in our study. Therefore, the shorter RR interval should be interpreted as a difference in resting heart rate rather than as direct evidence of increased sympathetic activity.

Beyond conventional QT measurements, Tp-e-based indices have been proposed as complementary markers for characterising ventricular repolarisation heterogeneity [7,15]. The Tp-e interval has traditionally been considered an electrocardiographic index related to dispersion of ventricular repolarisation, while the Tp-e/QT ratio expresses this interval relative to the overall duration of ventricular repolarisation [7,15]. Previous studies have associated increased Tp-e and Tp-e/QT values with ventricular arrhythmic events in selected high-risk populations, including patients with long QT and Brugada syndromes [8,16]. In the present study, Tp-e did not differ significantly between morbidly obese individuals and controls, whereas Tp-e/QT was higher in the primary between-group comparison but did not remain statistically significant after multiple-comparison correction. Accordingly, our findings do not support interpreting an isolated increase in Tp-e/QT as evidence of increased clinical arrhythmic risk in morbid obesity. Rather, Tp-e-based indices should be considered complementary surrogate measures of ventricular repolarisation, the clinical significance of which requires confirmation using longitudinal rhythm monitoring and arrhythmic outcomes.

Sex-related differences were observed in several Tp-e-based indices in the present study. Within the morbidly obese group, men had higher Tp-e, Tp-e/QTcB, Tp-e/QTcF, and Tp-e/QT values than women, and these differences remained statistically significant after correction for multiple comparisons. Sex-related differences in ventricular repolarisation are well recognised and have been attributed, at least partly, to the effects of sex hormones on cardiac ion-channel function and repolarisation dynamics [17,18,19]. Testosterone, in particular, has been suggested to modulate ventricular repolarisation through effects on calcium and potassium currents [17,18]. However, testosterone and other sex hormone concentrations were not measured in the present study, precluding assessment of a hormonal mechanism. Moreover, the obesity-by-sex interaction was not statistically significant, suggesting that the association between obesity and Tp-e/QT did not differ significantly between men and women. Therefore, the observed sex differences should be interpreted as sex-related variation in ventricular repolarisation within the morbidly obese group rather than evidence of a sex-specific effect of obesity.

An additional exploratory finding of the present study was the association between iron metabolism parameters and ventricular repolarisation indices within the morbidly obese group. Several correlations involving ferritin, transferrin saturation, and total iron-binding capacity remained significant after correction for multiple comparisons, with ferritin–Tp-e/QT showing the largest correlation coefficient among the FDR-significant ferritin-related associations. This association was subsequently evaluated in a post hoc exploratory multivariable model. After logarithmic transformation of ferritin, the positive association between ln-ferritin and Tp-e/QT remained significant after adjustment for age, sex, NLR, and glucose. The relationship between iron metabolism and ventricular repolarisation, however, appears to be complex. Previous studies have reported increased Tp-e, Tp-e/QT, and Tp-e/QTc values in individuals with low iron stores, suggesting that iron deficiency may also be associated with alterations in ventricular repolarisation [20]. In obesity, interpretation of ferritin is particularly challenging because ferritin reflects not only body iron stores but also inflammatory and metabolic processes and may be elevated as an acute-phase reactant [21]. Therefore, the positive association between ferritin and Tp-e/QT observed in our study should not be interpreted as evidence of iron overload or myocardial iron deposition. Rather, ferritin may represent a composite marker of iron status, inflammation, and metabolic disturbance in morbid obesity. The association remained statistically significant after adjustment for age, sex, NLR, and glucose; however, residual confounding by other measured or unmeasured factors cannot be excluded. Accordingly, the iron-related findings should be considered exploratory and hypothesis-generating rather than confirmatory, and further studies incorporating more comprehensive iron-status assessment, inflammatory markers, and, where appropriate, direct measures of tissue iron are required to clarify the relationship between iron metabolism and ventricular repolarisation in morbid obesity.

Our study has certain limitations. First, its retrospective, single-centre design and relatively small sample size may limit the generalisability of the findings. Second, long-term clinical follow-up for ventricular arrhythmias and advanced rhythm monitoring, such as Holter monitoring or telemetry, were not available. Therefore, the electrocardiographic parameters evaluated in the present study should be regarded as surrogate indices of ventricular repolarisation rather than direct measures of arrhythmic risk, and no conclusions regarding the incidence or prognosis of clinical arrhythmias can be drawn from these findings. Third, ECG measurements were obtained from a single lead (V6), which may introduce lead-dependent variability. Intraobserver reproducibility was assessed in a randomly selected subset of ECG recordings and showed a high level of agreement for both QT and Tp-e measurements. However, interobserver reproducibility was not assessed. Therefore, the use of a single lead and the absence of interobserver reproducibility assessment remain methodological limitations. Fourth, the use of a comparatively healthy non-obese control group may have introduced a healthy-control selection effect because the comorbidity burden differed substantially between the groups. Although a sensitivity analysis restricted to morbidly obese individuals without recorded comorbidities was performed to address this issue, the smaller size of this subgroup may have reduced statistical power. Furthermore, the covariates included in the multivariable models cannot account for all obesity-related clinical and metabolic factors, and residual confounding by unmeasured or incompletely characterised factors, including blood pressure, obstructive sleep apnoea, glycaemic status, inflammatory status, and medication exposure, cannot be excluded. Fifth, sex hormone concentrations, including testosterone, were not available; therefore, the mechanisms underlying the observed sex-related differences in repolarisation indices could not be directly investigated. Finally, the analyses of iron metabolism parameters were exploratory. Ferritin may be influenced by both iron status and inflammation, and direct measures of myocardial or tissue iron were not available; therefore, the observed associations should not be interpreted as evidence of iron overload or myocardial iron deposition.

The study also has several strengths. Ventricular repolarisation was evaluated using multiple complementary ECG indices, including QT, Tp-e, Tp-e/QT, and Tp-e/QTc, and QT correction was assessed using both the Bazett and Fridericia formulae. The use of the Fridericia formula was particularly relevant given the between-group difference in RR interval and allowed assessment of the robustness of the QTc findings to the method of heart-rate correction. In addition, multiple-comparison correction, effect-size estimation, and multivariable analyses were incorporated to provide a more conservative assessment of the observed associations. Sensitivity analyses addressing differences in comorbidity burden and the potential influence of heart-rate-related differences provided additional assessments of the robustness of the principal findings. All ECG measurements were performed by the same investigator using a standardised measurement protocol. Finally, the exploratory evaluation of sex-related differences and iron metabolism parameters alongside conventional repolarisation indices provides hypothesis-generating data on factors that may contribute to interindividual variation in ventricular repolarisation among individuals with morbid obesity.

## 5. Conclusions

The present findings do not support a consistent association between morbid obesity alone and altered ventricular repolarisation, as the observed between-group differences were not robust across multiple-comparison correction, adjusted analyses, and sensitivity analyses. Sex-related differences were observed in several Tp-e-based indices, suggesting that individual clinical characteristics may contribute to interindividual variation in ventricular repolarisation. The associations observed with iron metabolism parameters should be regarded as exploratory and hypothesis-generating. Overall, these findings highlight the multifactorial nature of ventricular repolarisation in morbid obesity and warrant prospective studies incorporating longitudinal rhythm monitoring and clinical arrhythmic outcomes.

## Figures and Tables

**Table 1:** Demographic and anthropometric characteristics of the morbidly obese group and the control group.

	Morbidly Obese Group (n = 112)	Control Group (n = 51)	*p*
**Age (years)**	36.37 ± 9.92 ^a^	36.39 ± 12.12 ^a^	0.988 ^b^
Sex (n,%)			
Male	33 (29.5%)	15 (29.4%)	0.995 ^c^
Female	79 (70.5%)	36 (70.6%)	
Height (metres)	1.64 ± 0.10 ^a^	1.64 ± 0.07 ^a^	0.981 ^b^
Body weight (kg)	120.10 ± 19.71 ^a^	76.10 ± 17.80 ^a^	<0.001 ^b,^*
BMI (kg/m^2^)	45.36 ± 3.03 ^a^	24.13 ± 3.47 ^a^	<0.001 ^b,^*

^a^: Mean ± Standard Deviation, ^b^: Independent *t*-test, ^c^: Chi-square test. *: Statistically significant (*p* < 0.05). BMI: Body Mass Index.

**Table 2:** Comorbid conditions in morbidly obese patients.

	Morbidly Obese Group (n = 112)
Comorbid conditions	67 (59.8%)
Hypertension	35 (31.2%)
Diabetes mellitus	19 (17%)
Dyslipidaemia	13 (11.6%)
Hypothyroidism	10 (8.9%)
Asthma/COPD/OSA	10 (8.9%)

COPD: Chronic Obstructive Pulmonary Disease, OSA: Obstructive Sleep Apnea.

**Table 3:** Comparison of biochemical parameters between the morbidly obese group and the control group.

	Morbidly Obese Group (n = 112)	Control Group (n = 51)	*p*
Glucose (mg/dL)	100 [90.02–111.75] ^a^	88 [84–94] ^a^	<0.001 ^c,^*
Magnesium (mg/dL)	1.97 [1.90–2.08] ^a^	1.91 [1.85–2.01] ^a^	0.023 ^c,^*
Leukocytes (10^3^/µL)	8.37 [6.97–9.75] ^a^	6.99 [6.31–8.64] ^a^	0.001 ^c,^*
Neutrophils (10^3^/µL)	5.28 [4.04–6.29] ^a^	3.86 [3.29–5.20] ^a^	<0.001 ^c,^*
Lymphocytes (10^3^/µL)	2.42 ± 0.70 ^b^	2.43 ± 0.69 ^b^	0.954 ^d^
Haemoglobin (g/dL)	13.69 ± 1.79 ^b^	13.18 ± 1.38 ^b^	0.078 ^d^
NLR	2.20 [1.64–2.81] ^a^	1.72 [1.34–2.05] ^a^	<0.001 ^c,^*

^a^: Median [Interquartile Range], ^b^: Mean ± Standard Deviation, ^c^: Mann-Whitney U test, ^d^: Independent *t*-test. *: Statistically significant (*p* < 0.05). NLR: Neutrophil-to-lymphocyte ratio.

**Table 4:** Comparison of electrocardiographic ventricular repolarisation parameters between the morbidly obese and control groups.

	Morbidly Obese Group (n = 112)	Control Group (n = 51)	Raw *p*	FDR-Adjusted *p*	Effect Size (95% CI)
RR (ms)	732.20 ± 128.25 ^a^	781.15 ± 109.93 ^a^	0.020 **^c^**	0.080	Hedges’ g = −0.397 (−0.746 to −0.092)
QT (ms)	344.20 [327.30–367.20] ^b^	349.20 [337.20–369.20] ^b^	0.380 ^d^	0.436	r_rb = −0.086 (−0.269 to 0.098)
QTc-Bazett (ms)	407.12 [394.1–424.02] ^b^	398.35 [384.1–415.21] ^b^	0.016 ^d^	0.080	r_rb = 0.236 (0.048 to 0.424)
QTc-Fridericia (ms)	384.72 [371.46–404.60] ^b^	382.72 [363.91–396.27] ^b^	0.240 ^d^	0.429	r_rb = 0.115 (−0.077 to 0.299)
Tp-e (ms)	77.40 [68.40–87.10] ^b^	74.80 [69.20–84.40] ^b^	0.268 ^d^	0.429	r_rb = 0.108 (−0.082 to 0.293)
Tp-e/QTc-Bazett	0.193 [0.172–0.212] ^b^	0.188 [0.175–0.212] ^b^	0.816 ^d^	0.816	r_rb = 0.023 (−0.169 to 0.212)
Tp-e/QTc-Fridericia	0.201 [0.184–0.224] ^b^	0.197 [0.182–0.217] ^b^	0.381 ^d^	0.436	r_rb = 0.086 (−0.101 to 0.273)
Tp-e/QT	0.229 [0.209–0.251] ^b^	0.214 [0.202–0.231] ^b^	0.030 ^d^	0.080	r_rb = 0.212 (0.025 to 0.393)

^a^: Mean ± Standard Deviation, ^b^: Median [Interquartile Range], ^c^: Independent *t*-test, ^d^: Mann-Whitney U test. To account for multiple comparisons, *p*-values were adjusted using the Benjamini–Hochberg false discovery rate (FDR) procedure. Hedges’ g was used as the standardized effect size for RR, and rank-biserial correlation (r_rb) was used for Mann–Whitney U comparisons. Confidence intervals for effect sizes were estimated using percentile bootstrap with 10,000 resamples performed independently within groups with replacement. Positive r_rb values indicate higher values in the morbidly obese group, whereas negative values indicate higher values in the control group. CI: confidence interval; QTc: corrected QT interval; Tp-e: T-wave peak-to-end interval.

**Table 5:** Sensitivity analysis of electrocardiographic ventricular repolarisation parameters in morbidly obese individuals without recorded comorbidities and healthy controls.

	Morbidly Obese Without Recorded Comorbidities (n = 45)	Control Group (n = 51)	Raw *p*	FDR-Adjusted *p*	Effect Size (95% CI)
RR (ms)	721.50 ± 138.53 ^a^	781.15 ± 109.93 ^a^	0.021 **^c^**	0.168	Hedges’ g = -0.477 (-0.939 to -0.072)
QT (ms)	338.40 [321.40–362] ^b^	349.20 [337.20–369.20] ^b^	0.082 ^d^	0.210	r_rb = -0.206 (-0.436 to 0.027)
QTc-Bazett (ms)	406.39 [392.07–425.76] ^b^	398.35 [384.11–415.21] ^b^	0.105 ^d^	0.210	r_rb = 0.192 (-0.040 to 0.417)
QTc-Fridericia (ms)	381.76 [370.65–398.13] ^b^	382.72 [363.91–396.27] ^b^	0.846 ^d^	0.846	r_rb = 0.023 (-0.208 to 0.260)
Tp-e (ms)	76 [68–85.80] ^b^	74.80 [69.20–84.40] ^b^	0.803 ^d^	0.846	r_rb = 0.030 (-0.203 to 0.264)
Tp-e/QTc-Bazett	0.186 [0.168–0.204] ^b^	0.188 [0.175–0.212] ^b^	0.829 ^d^	0.846	r_rb = -0.026 (-0.263 to 0.204)
Tp-e/QTc-Fridericia	0.201 [0.184–0.224] ^b^	0.197 [0.182–0.217] ^b^	0.689 ^d^	0.846	r_rb = 0.047 (-0.187 to 0.279)
Tp-e/QT	0.199 [0.183–0.220] ^b^	0.214 [0.202–0.231] ^b^	0.066 ^d^	0.210	r_rb = 0.218 (-0.011 to 0.441)

^a^: Mean ± Standard Deviation, ^b^: Median [Interquartile Range], ^c^: Independent *t*-test, ^d^: Mann-Whitney U test. To account for multiple comparisons, *p*-values were adjusted using the Benjamini–Hochberg false discovery rate (FDR) procedure. Hedges’ g was used as the standardized effect size for RR, and rank-biserial correlation (r_rb) was used for Mann–Whitney U comparisons. Confidence intervals for effect sizes were estimated using percentile bootstrap with 10,000 resamples performed independently within groups with replacement. Positive r_rb values indicate higher values in the morbidly obese group, whereas negative values indicate higher values in the control group. CI: confidence interval; QTc: corrected QT interval; Tp-e: T-wave peak-to-end interval.

**Table 6:** Comparison of electrocardiographic ventricular repolarisation parameters by sex in the morbidly obese group.

	Male (n = 33)	Female (n = 79)	Raw *p*	FDR-Adjusted *p*	Effect Size (95% CI)
RR (ms)	698.87 ± 139.22 ^a^	746.11 ± 121.60 ^a^	0.075 ^c^	0.121	Hedges’ g = −0.370 (−0.818 to 0.057)
QT (ms)	333.60 [320.20–364.40] ^b^	352.40 [329.60–368] ^b^	0.234 ^d^	0.269	r_rb = −0.143 (−0.391 to 0.115)
QTc-Bazett (ms)	411.48 [394.86–436.15] ^b^	406.91 [392.05–422.14] ^b^	0.185 ^d^	0.244	r_rb = 0.160 (−0.077 to 0.395)
QTc-Fridericia (ms)	383.12 [370.60–411.61] ^b^	385.03 [371.64–404.38] ^b^	0.762 ^d^	0.764	r_rb = 0.036 (−0.209 to 0.283)
Tp-e (ms)	84.80 [73.80–97.60] ^b^	76.80 [68–84] ^b^	0.012 ^d^	0.031 *	r_rb = 0.302 (0.058 to 0.532)
Tp-e/QTc-Bazett	0.20 [0.17–0.23] ^b^	0.18 [0.17–0.20] ^b^	0.015 ^d^	0.031 *	r_rb = 0.292 (0.038 to 0.531)
Tp-e/QTc-Fridericia	0.21 [0.19–0.25] ^b^	0.19 [0.18–0.21] ^b^	0.004 ^d^	0.017 *	r_rb = 0.345 (0.100 to 0.568)
Tp-e/QT	0.23 [0.22–0.27] ^b^	0.21 [0.20–0.24] ^b^	<0.001 ^d^	0.001 *	r_rb = 0.465 (0.246 to 0.666)

^a^: Mean ± Standard Deviation, ^b^: Median [Interquartile Range], ^c^: Independent *t*-test, ^d^: Mann-Whitney U test. To account for multiple comparisons within this exploratory sex-based analysis, *p*-values were adjusted using the Benjamini–Hochberg false discovery rate (FDR) procedure. Hedges’ g was used as the standardized effect size for RR, and rank-biserial correlation (r_rb) was used for Mann–Whitney U comparisons. Confidence intervals for effect sizes were estimated using percentile bootstrap with 10,000 resamples performed independently within groups with replacement. Positive r_rb values indicate higher values in males, whereas negative values indicate higher values in females. *: Statistically significant (FDR-adjusted *p* < 0.05). CI: confidence interval; QTc: corrected QT interval; Tp-e: T-wave peak-to-end interval.

**Table 7:** Multivariable linear regression analysis of factors associated with Tp-e/QT ratio.

Variable	B	95% CI for B	Standardized β	*p*
Morbid obesity (vs control)	0.006	−0.006 to 0.018	0.077	0.338
Female sex (vs male)	**−0.024**	**−0.039 to −0.010**	−0.335	0.001 *
Age	0.000	0.000 to 0.001	0.138	0.085
Magnesium	−0.005	−0.045 to 0.035	−0.022	0.799
Potassium	−0.003	−0.020 to 0.014	−0.028	0.727
Creatinine	0.015	−0.036 to 0.066	0.061	0.566
Calcium	−0.001	−0.017 to 0.015	−0.008	0.928

**Model statistics:** R^2^ = 0.169; adjusted R^2^ = 0.126; F(7,137) = 3.977; *p* = 0.001. B, unstandardized regression coefficient; β, standardized regression coefficient; CI, confidence interval. Morbid obesity was coded as 1 and control as 0; male sex as 0 and female sex as 1. The analysis was performed using complete cases. *: Statistically significant (*p* < 0.05).

**Table 8:** Correlations between iron metabolism parameters and electrocardiographic ventricular repolarisation indices in the morbidly obese group.

Iron Parameter	ECG Parameter	Correlation Coefficient	Raw *p*	FDR-Adjusted *p*
Serum iron	RR	0.083 ^a^	0.386	0.562
	QT	0.080 ^a^	0.404	0.562
	QTc-Bazett	0.046 ^a^	0.632	0.697
	Tp-e	0.189 ^a^	0.048	0.092
	Tp-e/QTc-Bazett	0.207 ^a^	0.030	0.074
	Tp-e/QT	0.188 ^a^	0.049	0.092
	QTc-Fridericia	0.075 ^a^	0.435	0.581
	Tp-e/QTc-Fridericia	0.196 ^a^	0.040	0.092
TIBC	RR	−0.093 ^b^	0.336	0.512
	QT	−0.070 ^a^	0.474	0.589
	QTc-Bazett	−0.061 ^a^	0.528	0.623
	Tp-e	−0.271 ^a^	**0.005**	**0.026** *
	Tp-e/QTc-Bazett	−0.309 ^a^	**0.001**	**0.011** *
	Tp-e/QT	−0.269 ^a^	**0.005**	**0.026** *
	QTc-Fridericia	−0.069 ^a^	0.479	0.589
	Tp-e/QTc-Fridericia	−0.304 ^a^	**0.001**	**0.011** *
Transferrin saturation	RR	0.120 ^a^	0.226	0.381
	QT	0.048 ^a^	0.627	0.697
	QTc-Bazett	−0.009 ^a^	0.930	0.991
	Tp-e	0.188 ^a^	0.056	0.100
	Tp-e/QTc-Bazett	0.244 ^a^	**0.013**	**0.045** *
	Tp-e/QT	0.196 ^a^	0.046	0.092
	QTc-Fridericia	0.005 ^a^	0.962	0.993
	Tp-e/QTc-Fridericia	0.232 ^a^	0.018	0.052
Ferritin	RR	−0.240 ^a^	**0.013**	**0.045** *
	QT	−0.235 ^a^	**0.015**	**0.047** *
	QTc-Bazett	0.000 ^a^	0.999	0.999
	Tp-e	0.219 ^a^	0.023	0.061
	Tp-e/QTc-Bazett	0.260 ^a^	**0.006**	**0.030** *
	Tp-e/QT	0.386 ^a^	**<0.001**	**0.001** *
	QTc-Fridericia	−0.097 ^a^	0.317	0.508
	Tp-e/QTc-Fridericia	0.310 ^a^	**0.001**	**0.011** *

^a^: Spearman’s correlation, ^b^: Pearson’s correlation. To account for multiple comparisons across the 32 exploratory iron–ECG correlations, *p*-values were adjusted using the Benjamini–Hochberg false discovery rate (FDR) procedure. Pairwise available observations were used; therefore, sample sizes varied according to missing laboratory data. *: Statistically significant (FDR-adjusted *p* < 0.05). TIBC: total iron-binding capacity; QTc: corrected QT interval; Tp-e: T-wave peak-to-end interval.

## Data Availability

Dataset available on request from the authors.
